# Microencapsulation of *Bacillus* Strains for Improving Wheat (*Triticum turgidum Subsp. durum*) Growth and Development

**DOI:** 10.3390/plants11212920

**Published:** 2022-10-29

**Authors:** Jonathan Rojas-Padilla, Luz Estela de-Bashan, Fannie Isela Parra-Cota, Jorge Rocha-Estrada, Sergio de los Santos-Villalobos

**Affiliations:** 1 Instituto Tecnológico de Sonora, 5 de Febrero 818 sur, Ciudad Obregon 85000, Sonora, Mexico; 2 The Bashan Institute of Science, 1730 Post Oak Court, Auburn, AL 36830, USA; 3 Environmental Microbiology Group, Northwestern Center for Biological Research (CIBNOR), Av. IPN 195, La Paz 23096, Baja California Sur, Mexico; 4 Department of Entomology and Plant Pathology, Auburn University, 301 Funches Hall, Auburn, AL 36849, USA; 5 Campo Experimental Norman E. Borlaug, Instituto Nacional de Investigaciones Forestales, Agrícolas y Pecuarias, Ciudad Obregon 85000, Sonora, Mexico; 6 CONACyT Unidad Regional Hidalgo, Centro de Investigación en Alimentación y Desarrollo, Pachuca Ciudad del Conocimiento y la Cultura, San Agustín Tlaxiaca 42163, Hidalgo, Mexico

**Keywords:** PGPR, microbial inoculant, alginate, microbeads, *Bacillus*, wheat

## Abstract

Bio-formulation technologies have a limited impact on agricultural productivity in developing countries, especially those based on plant growth-promoting rhizobacteria. Thus, calcium alginate microbeads were synthesized and used for the protection and delivery of three beneficial *Bacillus* strains for agricultural applications. The process of encapsulation had a high yield per gram for all bacteria and the microbeads protected the *Bacillus* strains, allowing their survival, after 12 months of storage at room temperature. Microbead analysis was carried out by observing the rate of swelling and biodegradation of the beads and the released-establishment of bacteria in the soil. These results showed that there is an increase of around 75% in bead swelling on average, which allows for larger pores, and the effective release and subsequent establishment of the bacteria in the soil. Biodegradation of microbeads in the soil was gradual: in the first week, they increased their weight (75%), which consistently results in the swelling ratio. The co-inoculation of the encapsulated strain TRQ8 with the other two encapsulated strains showed plant growth promotion. TRQ8 + TRQ65 and TRQ8 + TE3^T^ bacteria showed increases in different biometric parameters of wheat plants, such as stem height, root length, dry weight, and chlorophyll content. Thus, here we demonstrated that the application of alginate microbeads containing the studied strains showed a positive effect on wheat plants.

## 1. Introduction

Wheat is one of the three major kinds of cereal (together with maize and rice) and contributes as a source of energy, protein, and fiber to the human diet [1]. It is a staple food crop for more than one-third of the world’s population [2]. In Mexico, wheat is one of the most important fertilized and irrigated crops, occupying half a million hectares in 2018. At present, ~30% of the national wheat production is cultivated in the Yaqui Valley, located in northwestern Mexico [3]. This valley, with a semiarid climate, a mean annual temperature of 24.7 °C, a rainfall of 320 mm year^−1^, and evapotranspiration of 2000 mm year^−1^ [4], is one of the most intensive agricultural regions of the world, by using irrigation water, fertilizers, and constantly improving cultivars for one of the highest yields globally [5,6]. Thus, this region is characterized, for example, by the application of high doses of chemical fertilizers, mainly nitrogen (~300 kg N ha^−1^) [7]. This results in significant N losses through leaching and volatilization that cause economic and environmental problems, such as N_2_O emissions, eutrophication, and loss of soil fertility [4,8]. Hence, there is an immediate need to find alternative strategies for increasing the wheat yield, while maintaining the long-term ecological balance in the agroecosystem.

Plant growth-promoting rhizobacteria (PGPR) have been used for decades to increase crop yield; these bacteria can improve the health and quality of plants by stimulating plant growth through direct and/or indirect mechanisms. Direct mechanisms include siderophore production, phosphorus solubilization, enzyme production (ACC deaminase, cellulases, and chitinases), phytohormone production (auxins, gibberellins, and cytokinins), and fixing atmospheric nitrogen [9,10,11,12]. Indirect mechanisms are related to plant protection against abiotic (salinity, drought, and temperature) and biotic (phytopathogens) agents by induction of stress resistance, lytic enzymes, toxins, siderophores, biosurfactants, and activation of defensive signaling [13,14,15,16].

Several bacterial species have been reported as PGPR, which belong to the genus *Azospirillum*, *Pseudomonas*, *Azotobacter*, *Burkholderia*, *Paenibacillus*, *Acinetobacter*, *Rhizobium*, *Stenotrophomonas*, *Serratia*, *Flavobacterium*, *Jeotgalicoccus*, and *Bacillus* [8,17,18]. Additionally, the genus *Bacillus* has other advantageous traits, such as the ability to form endospores [13], metabolism of complex sources of essential nutrients [19], iron chelating properties by the production of siderophores [13], and production of plant growth-promoting and biocontrol metabolites. The impact of these mechanisms on crop growth and yield has been widely reported, i.e., increasing the Phosphorus (P) and N uptake by roots of maize and oriental melon when plants were inoculated with *Bacillus* sp. LKE15 and *Bacillus pumilus* S1r1 [19,20]; B. megaterium BcM33 increased the dry weight of the cotton (*Gossypium hirsutum*) root and stem by 11% and 18%, respectively [21]; *B. pumilus* RS3 inoculated in soybean seeds enhanced the plant growth and increased seed protein yield [22]; *B. subtilis* 21-1 has been reported as both plant growth promoter and disease suppressor in association with cucumber (*Cucumis sativus* L.), Chinese cabbage (*Brassica rapa Subsp. pekinensis*), lettuce (*Lactuca sativa*) and tomato (*Lycopersicon esculentum* Miller) [23,24].

The positive effect of inoculated PGPR on growth and yield in crops still requires several issues to be addressed, i.e., the bacterial viability, plant and/or soil colonization by inoculated strains, and microbial biosynthesis of active metabolites involved in the plant growth regulation [11]. Thus, diverse strategies have been developed for improving the viability and colonization of inoculated PGPR under different soil and climatic conditions in agroecosystems; among these, microencapsulation has been proposed as an alternative to increasing the shelf life of microbial inoculants [10,25,26,27]. This technology creates a wall-like barrier that permits (i) a controlled release of encapsulated microorganisms for successful root colonization; (ii) provisional physical protection of inoculated microorganisms against microbial competitors, stressful environmental conditions, and mini-predators; and (iii) the polymeric carrier is slowly degraded by native soil microbiota for releasing the immobilized microorganism [28,29]. Thus, the objective of this research work was to quantify the shelf life of microencapsulated *Bacillus* strains (optimizing the sodium alginate and CaCl_2_ concentration), as well as the biodegradation capacity of the obtained microbeads, and their growth-promoting effect on wheat, under greenhouse conditions.

## 2. Results

### 2.1. Viability of Studied Strains Exposed to Sodium Alginate

**Figure 1:**
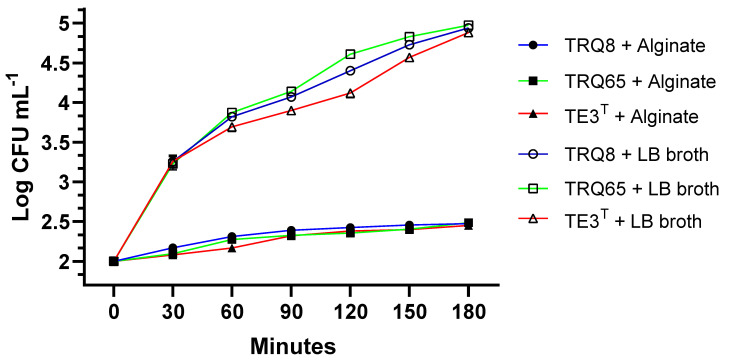
Growth kinetics in sodium alginate, and LB Broth of (●) *Bacillus megaterium* TRQ8, (■) *Bacillus paralicheniformis* TRQ65, and (▲) *Bacillus cabrialesii* TE3^T^, growing at 32 °C, and 150 rpm. Bars represent the standard deviation.

To assess the viability of PGPR strains in the candidate encapsulation compound, a concentration of 2% sodium alginate was used. Growth curves were performed and measured the doubling time (t_d_) of these bacteria, growing in sodium alginate (as the sole carbon source) and in LB broth as a control. For strain TRQ65, we found a t_d_ = 0.30 h in LB broth and t_d_ = 1.86 h in sodium alginate; strain TRQ8 and TE3^T^ had a doubling time of 1.91 in LB and 2.01 h in 2% sodium alginate, respectively, while the doubling time in LB broth was 0.31 h for both strains (Figure 1). Our results show that even when the doubling time of all strains increased in sodium alginate with respect to LB, they were still capable of growing using this polymer as a carbon source. Hence, sodium alginate was used as encapsulation material.

### 2.2. Alginate Encapsulation of PGPR Strains and Capsule Shelf

CFU counts were assessed before and after encapsulation, to calculate the encapsulation yield. As shown in Table 1, once the encapsulation process was done, approximately 18 g of dry microbeads (<200 µm) containing each studied *Bacillus* strain was recovered, obtaining a sodium alginate yield of ~90% and a CFU yield of ~ 4.7 × 10^11^ CFU·g^−1^ on average (Table 1). From these values, we calculated the highest encapsulation yield (Equation (1)) of 338.0% was for *Bacillus paralicheniformis* TRQ65 and the lowest for *B. megaterium* TRQ8 with a yield of 133.9%.

**Figure 2:**
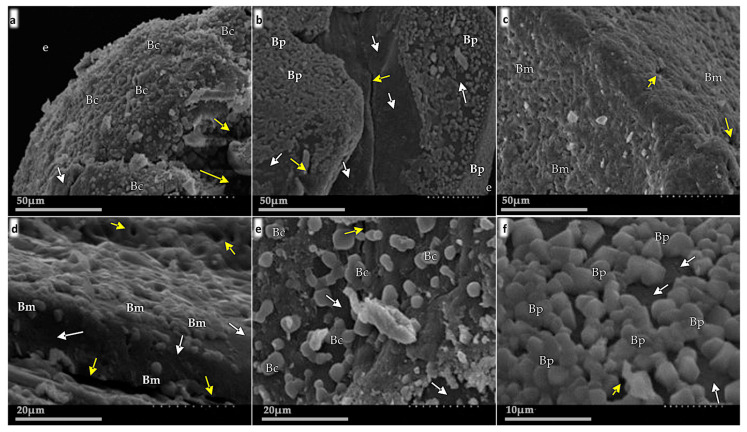
Scanning electron microscopy (SEM) of the surface of microbeads, where white arrows indicate the surface of the microbead, and yellow arrows indicate pores. (**a**) *Bacillus cabrialesii* TE3^T^ *(Bc)* microbead rim. (**b**) Microbead containing *B. paralicheniformis* TRQ65 *(Bp)*, showing the space and structure of the internal cavity (typical of alginate microbeads) without bacteria, and the sides loaded with bacteria. (**c**) Shows the surface of a microbead containing *B. megaterium* TRQ8 (*Bm)*. (**d**) Magnification and side view of a microbead containing *B. megaterium* TRQ8, showing more clearly a load of this strain and its distribution in the microbead. (**e**) Magnification of subfigure (**a**) showing the population of *B. cabrialesii* on the microbead. (**f**) Close-up of the subfigure (**b**) showing the microbead surface colonized by *B. paralicheniformis*.

SEM micrographs showed the presence of each studied strain on the surface and the interior of obtained microbeads (Figure 2). The internal layer of microbeads had pore-like microstructures with a small diameter (~2 µm) (Figure 2; yellow arrows), and a closer examination of these structures showed that they contain bacterial cells (Figure 2d,e).

**Figure 3:**
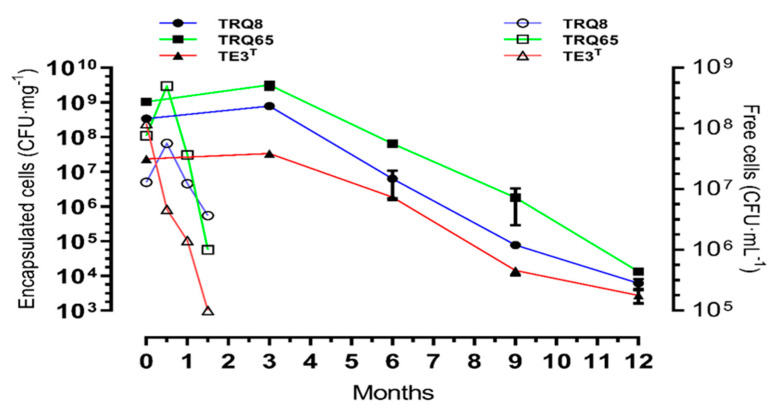
Storage trial of encapsulated (●) *Bacillus megaterium* TRQ8, (■) *B. paralicheniformis* TRQ65, and (▲) *B. cabrialesii* TE3^T^ and free cells (○) *B. megaterium* TRQ8, (□) *B. paralicheniformis* TRQ65, and (∆) *B. cabrialesii* TE3^T^. Bars indicate standard deviation.

In the storage test (20–25 °C for 12 months), the bacterial concentration in microbeads during the first 3 months did not show significant differences (3.8 × 10^9^, 7.83 × 10^8,^ and 3.36 × 10^7^ CFU·mg^−1^ of strain TRQ65, TRQ8, and TRQ65, respectively) (Figure 3). After 3 months, CFU decreased gradually until the twelfth month, where the bacterial population in microbeads reached 1.0 × 10^5^, 8.0 × 10^4^, and 3.3 × 10^5^ CFU·mg^−1^ of strain TE3^T^, TRQ8, and TRQ65, respectively (Figure 3).

### 2.3. Swelling Studies

**Figure 4:**
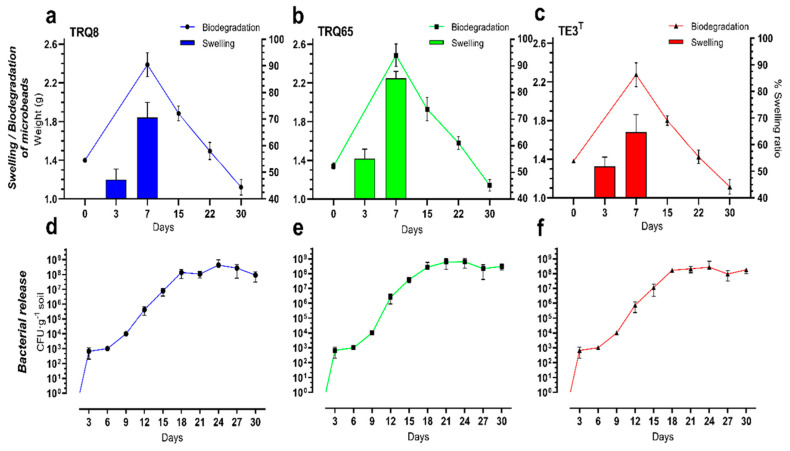
Biodegradation and swelling ratio of microbeads (**a**–**c**) and *Bacillus* strains establishment (**d**–**f**) in soil. Bars indicate the standard deviation.

The percentage of swelling ratio (SR) for each formulation (Figure 4a–c) in the first three days was 51.4% on average. Strain TRQ65 microbeads had the highest swelling with 85.3% after one week in water-saturated soil. The SR of strain TE3^T^ microbeads was the lowest (64.6%), while strain TRQ8 showed 70.5% in one week. All microbeads similarly increased swelling, this indicates they adsorb similar amounts of water from the environment; water adsorption is crucial for the metabolic activity of cells before the degradation of the microbeads and release of the strains in soil.

### 2.4. Biodegradation of Microbeads and Establishment of Released Studied Bacteria in the Soil

The biodegradation of microbeads in the soil was gradual. This assay started with 1 g of microbeads + nylon bag weight (~0.373 g). Initially, we observed an increase in weight (75% on average) for microbeads containing each encapsulated strain (Figure 4). This is consistent with the swelling behavior, due to the increase in water uptake in the first week (average of 73.4%, Figure 4). Subsequently, and due to desiccation/degradation of microbeads, the weight gradually decreased after 2 weeks until day 30, with the lowest value for strain TRQ65 with 14.8% less, strain TE3^T^ and TRQ8 with greater biodegradation with 19.2%, and 19.8% less weight, respectively (Figure 4a–c).

On the other hand, the establishment of released bacterial strains in soil showed an exponential increase in the first 15 days (Figure 4d–f); in this phase, the doubling time (t_d_) was 26.64, 25.56, and 22.68 h for strain TRQ8, TE3^T^, and TRQ65, respectively. In the next phase, from 15 to 30 days, the t_d_ increased to 101.1, 90.72, and 121.4 h, respectively. These results indicate that the bacterial cells established in the soil as follows: strain TRQ8 at 9.1 × 10^7^ CFU·g^−1^ of dry soil, strain TRQ65 at 3.1 × 10^8^ CFU·g^−1^ of dry soil, and strain TE3^T^ at 1.8 × 10^8^ CFU·g^−1^ of dry soil (Figure 4). Bacterial cells were undetected in the control (microbeads without bacteria), indicating that our experimental systems were axenic.

### 2.5. Effect of Encapsulated Studied Strains on Wheat Growth

**Figure 5:**
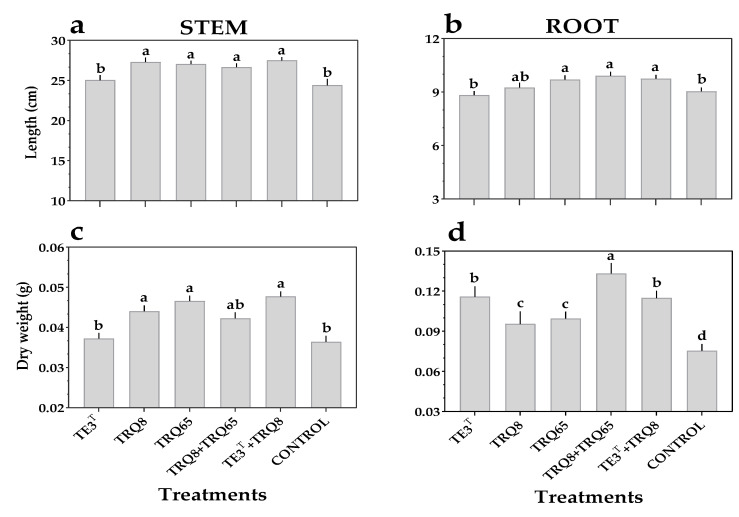
Wheat biometric effects by inoculation of *Bacillus* strains encapsulated in microbeads (individual or consortium) and control (empty microbeads) on (**a**) stem length, (**b**) root length, (**c**) stem dry weight and (**d**) root dry weight, after 30 days, under growth chamber conditions (13 h of darkness at 14 °C, 2 h of light at 18 °C, 7 h of light at 25 °C, and 2 h of light at 18 °C). Different letters correspond to treatments with significant differences according to Tukey HSD (*p* ≤ 0.05). Bars indicate standard error.

In growth chamber assays, wheat plants inoculated with encapsulated *Bacillus* strains exhibited a significant increase in biometric traits compared to un-inoculated plants (Figure 5). Encapsulated *B. paralicheniformis* TRQ65 induced a significant increase in stem (10.7%) and root (7.4%) length, while encapsulated *B. megaterium* TRQ8 increased the stem length by 11.8% and *B. cabrialesii* TE3^T^ showed a significant increase in root dry weight at 53.3%. Notably, co-inoculation of encapsulated strains (TRQ8 + TRQ65 or TE3^T^ + TRQ8) showed a significantly greater increase in stem and root length, compared to the control; in the case of TRQ8 + TRQ65, we found an increase of 9.2% and 9.7%, respectively; while TE3^T^ + TRQ8 increased stem length by 11.7% and root length by 7.9% (Figure 5a). For the stem dry weight, the individual inoculation of encapsulated strain TRQ8 and strain TRQ65, as well as the co-inoculation of strains TE3^T^ + TRQ8 showed the best results, with values greater than the control by 22.2%, 27.7%, and 30.5%, respectively (Figure 5b). For root dry weight, all treatments were significantly different from the control, as shown in Figure 5b, and the co-inoculation of TRQ8 + TRQ65 showed a better result, increasing the root dry weight by 77.3%; the individual inoculation of *B. cabrialesii* TE3^T^ also showed an increase of 53.3% in this trait, compared to un-inoculated plants (Figure 5b).

We also studied the growth promotion of wheat in greenhouse assays by the application of encapsulated bacteria (Table 2). In cycle 1 (December 2017–February 2018), the inoculation of encapsulated strain TRQ65 or strains TRQ8 + TRQ65 showed a significant increase in stem and root length, with an increase of 12.8% and 8.4% in stem length, respectively, and for root length, 15.1% and 19.2%, respectively, compared to the control. Inoculation of encapsulated strain TRQ8 and strains TE3^T^ + TRQ8 also showed a significant increase in root length, 17.5% and 15.8%, respectively. Stem dry weight and biovolume index (circumference × stem length) were significantly increased by the inoculation of encapsulated strain TRQ65, with values of 22.6% and 6.7% higher than the control, respectively. The co-inoculations of the microbeads (strains TE3^T^ + TRQ8 and strains TRQ8 + TRQ65) also had a slight positive effect on the stem dry weight, but without significant difference compared to un-inoculated plants. In the case of root dry weight, strains TE3^T^ + TRQ8 were significantly different from the control with an increase of 12.8%; similarly, the inoculation of strain TE3^T^ also showed positive changes in this measure (9.4%). Chlorophyll content in inoculated plants did not show statistically significant differences compared to the control treatment. In cycle 2 (December 2018–February 2019), the results showed maintenance of the metabolic capacities of the studied strains, while the same treatments and amount of microbeads (~0.02 g) were applied. Strain TRQ65 treatment maintained its growth-promoting capacity in variables such as root length and stem dry weight, which increased by 9.5% and 18.1%, respectively, showing a significant difference compared to the control. Strains TRQ8 + TRQ65 showed, as in the previous cycle, a positive significant difference in the length (stem and root) of 8.6% and 9%, respectively; in addition, in this second cycle, the root dry weight increased by 18.8% compared to the control. For chlorophyll content, the individual inoculations of strain TE3^T^ (5%) and TRQ8 (7.15%) were significantly different compared to the control, while co-inoculations of strains TRQ8 + TRQ65 and strains TE3^T^ + TRQ8 showed statistical difference compared to the control, increasing the chlorophyll content by ~7% in SPAD units. In the Biovolume index measurement, no statistical differences were induced by inoculations.

## 3. Discussion

In recent decades, new formulations have been exploited in agriculture for several purposes, including the application of plant-beneficial organisms, improving water use efficiency, enhancing seed germination, or delivering agrochemicals such as fertilizers or fungicides [30,31]. Bacterial strains in the present study have been reported as promising PGPR in studies of cells in a liquid formulation. *Bacillus cabrialesii* TE3^T^ was recently reported as a new species [32], and previous work demonstrated its metabolic promotion capabilities, such as phosphate solubilization, and biological control against *Bipolaris sorokiniana* [15]. Likewise, *B. megaterium* TRQ8 has shown the ability to increase growth in wheat seedlings, significantly improving biometric variables, such as root length and stem dry weight compared to un-inoculated plants [33]; furthermore, TRQ8 can produce indoles, solubilizes phosphate, and also shows siderophore production [34]. *B. paralicheniformis* TRQ65 showed indole production and has been reported as a plant growth promoter when the strain is co-inoculated with TRQ8, showing increases in wheat seedlings of stem length, root length, stem, and root dry weight, and biovolume index [34]. However, the identification of PGPR strains needs to be accompanied by novel strategies for their use. Thus, the development and application of innovative technologies for agriculture are crucial for supplying food to the growing human population.

In the earlier era of PGPR inoculants, liquid formulations, clays, and peat were used as carriers [35]. More recently, the application of a delivery system based on encapsulation technology represents a promising technique to store PGPR [36]. Alginate is one of the most widely used materials for encapsulation, due to its low cost, easy handling, and non-toxic nature [35,37], and so far, it seems to be the most promising material that has been tested [25,38]. However, even though alginate is non-toxic, studies on probiotics strains have found that it causes a decrease in the population of the microorganisms (~13% less of *Lactobacillus acidophilus* La-5 and ~19% less of *L. casei* 01) [37,39]. Hence, it is essential to test the survival of bacteria when they are in contact with sodium alginate. Since we did not find any decrease in the number of viable cells of our strains, we propose that the use of this polymer as an encapsulating material is appropriate.

The survival of encapsulated bacteria depends mainly on the encapsulation technique, the choice of the polymer, and the type of bacteria involved in the process. In addition, among different strains, there is a range of variability in responses to growth or stability [35]. In our study, bacteria were encapsulated with an optimal yield of 133.9% to 338.0%, the numbers are greater as compared to other encapsulation methods, i.e., *L. casei* encapsulated inside alginate-pectin microbeads with yields less than 79% [40], and a 75% encapsulation yield of *B. subtilis* B26 in microbeads of calcium alginate and pea protein [41]. In our study, the increase in the bacterial population during encapsulation is attributed to the procedure itself, because before encapsulating the bacterial suspension + sodium alginate, it is incubated for a period of 1 h and 24 h after the encapsulation for the drying of the microbeads (32 °C), considering that the bacteria in alginate medium have an average doubling time of ~1.92 h, sufficient time for bacterial reproduction. During the process of drying, the bacteria stay in a dormant form, i.e., their metabolism is very slow or even halted, and there is evidence that alginate plays a significant role in the stress protection mechanism for bacteria, as well as creating a hydrated microenvironment contributing to the biofilm architecture [36,42,43,44]. In previous studies, the highest mortality rate occurs shortly after encapsulating, while in storage, or following application, and the death rate reaches ~90% of the initial cell number before encapsulation during stress and storage [29,45]. Here, we achieved an initial concentration of 2.4 × 10^7^ CFU mg^−1^ for strain TE3^T^, 3.4 × 10^8^ CFU mg^−1^ for strain TRQ8, and 1.0 × 10^9^ CFU mg^−1^ for strain TRQ65 of bacteria in dry microbeads and the initial three months of storage at room temperature (~25 °C), the bacterial population per mg of dry microbeads increased 2.2%, 4.2%, and 5.3%, respectively. After one year of storage, ~45% reduction was observed for all studied strains but maintaining their plant growth promotion capabilities as was demonstrated by the greenhouse assay (cycle 2) (Table 2). The use of calcium alginate alone (not in microbeads) keeps the survival rate of *Pseudomonas striata* and *B. polymyxa* over an extended time at higher temperatures [46], and the encapsulation of probiotic bacteria increases the survival rate during storage, especially at higher temperatures [47,48]. Gonzalez et al. [27] reported that encapsulated *A. brasilense* maintained the same concentration in the first month, declined 5% after three months, and subsequently decreased by 76% seven months after storage at room temperature. The morphological traits of the *Bacillus* studied strains, and recovered from the microbeads were identical to those used in the encapsulation process and were supported by the macroscopic characteristics of each strain, related to its species [16,32,33]. Alginate beads are porous, which was characterized by SEM micrographs (Figure 2); it facilitated cell survival over long storage periods, protected bacterial cells against mechanical stress, and aided in the bacterial release of microbeads. Furthermore, the alginate is presumed to give nutrition in the form of ash, starch, protein, lipid, and sugar to the encapsulated cells [49,50]. All the attributes mentioned above together make this formulation a protector for bacterial cells and a stable microenvironment. 

We found that microbeads swelled when incorporated into the soil, as the water molecules spread in the compound’s network. He et al. [51] obtained similar swelling results in NaAlg microbeads immersed in a physiological saline solution. These results are consistent with those obtained in the biodegradation of the microbeads, wherein a mass increase was observed in the first week of analysis, and subsequently, the biodegradation was confirmed by the mass loss of the microbeads with prolonged incubation under simulated conditions of the Yaqui valley. According to Liakos et al. [52], the biodegradation of NaAlg occurred, probably through the breakdown of covalently linked (1-4) glycoside bonds of a polymer composed of unbranched chains β-dmannuronate and α-l-guluronate residues. Swapna et al. [53] found that degradation of *B. megaterium* beads (polymer + grain flours) in absence of a plant started on day 6 and complete degradation was observed on day 15 of incubation. Regarding the release of bacterial strains, the natural pores of alginate help bacterial release, and together with the expansion of the microbeads due to swelling, these pores grow, allowing the release of bacteria in the soil. The bacterial population levels in the soil in all treatments were maintained at threshold levels of 8 logs for three bacterial strains. A previous study demonstrated that sodium alginate improves the swelling ratio of microbeads, thus increasing the porosity and surface area of microbeads and enhancing the release of bacteria [28]. The results were consistent with the trend for biodegradability and swelling ratio (Figure 4a–c). Thereby, encapsulation allowed controlled and slow release from the alginate microbeads into the soil and facilitates the establishment of bacterial populations, since this filler material serves as a carbon source for bacteria [38].

The process of encapsulation did not change the nature of the *Bacillus* strains studied here, as their growth promotion capabilities, which were previously studied, were maintained. For example, wheat plants inoculated with free cells of *B. cabrialesii* TE3^T^, *B. megaterium* TRQ8, and *B. paralicheniformis* TRQ65 showed an increase compared to control, in chlorophyll content for strain TE3^T^, stem, and root dry weight for strain TRQ8 and stem length for strain TRQ65 [11,34]; here, the encapsulated strains also show a positive increase in the same biometric variables analyzed at different stages of the plant and adding improvements in the plant of chlorophyll content, root length, stem length, root dry weight and stem dry weight for the application of strain TRQ8 and strain TRQ65. The microbeads can protect PGPR applied to soil compared to bacterial suspension [54]. In other microencapsulation studies, a microbead formulation containing *A. brasilense* enhanced the development of wheat and mesquite seedlings by 55% and 40% of total dry weight, respectively [27,55]. Vassilev et al. [36] assure that the co-inoculation of immobilized microorganisms constitutes unexploited biotechnology for microbial formulation. 

The genus *Bacillus* has been widely reported as PGPR and can be used individually or in co-inoculation with other bacterial genera. In previous work, co-inoculations of the TRQ8 with the other two strains showed plant growth promotion [34], here the co-inoculation of *B. megaterium* TRQ8 and *B. paralicheniformis* TRQ65 showed an increase in length and dry weight, and content chlorophyll, as well as the co-inoculation of *B. cabrialessi* TE3^T^ and *B. megaterium* TRQ8 also showed increases in various biometric parameters of wheat plants, which suggests an increased mobilization of nutrients in the plant [56]. Various studies have shown that the capacities of the PGPR improve when they are inoculated in a consortium [57]. This may be due to several emergent interactions such as metabolic complementation, which can cause synergistic effects in the simultaneous use of the organic and inorganic compounds found in the soil [58]. In this study, phenotypic observations showed a growth-promoting effect induced by the presence of *Bacillus* microbeads close to wheat seedlings. This is probably due to the increased exposure of plants to microbeads. Altogether, the encapsulation of *Bacillus* strains did not appear to alter their growth-promoting behavior.

## 4. Materials and Methods

### 4.1. PGPR and Bacterial Growth Conditions

*Bacillus megaterium* TRQ8, *B. cabrialesii* TE3^T^, and *B. paralicheniformis* TRQ65 were obtained from the microbial collection named Coleccion de Microorganismos Edáficos y Endófitos Nativos (COLMENA, www.itson.edu.mx/COLMENA, accessed on 11 February 2019) [8,59]. These strains were isolated from wheat crop rhizosphere in commercial fields located in the Yaqui Valley (27°00′ and 28°00′ N and 109°30′ and 110°37′ W) and have been previously characterized for their PGP traits (at a genomic, metabolic, and functional level) [8,11,12,15,16,32,33,34,60].

For a routine culture of bacterial strains, frozen glycerol (−80 °C) stocks were streaked on Petri dishes containing LB agar (US Biological Cat. L1515) and incubated for 24 h at 35 °C. Later, an axenic colony of each strain was transferred individually into 10 mL of LB broth (US Biological Cat. L1520) contained in a conic tube (50 mL) and grown for 24 h at 35 °C, in a rotary shaker at 5× *g* (pre-inoculum). Five mL of each bacterial pre-inoculum (OD600 ~1.0) were used to inoculate 250 mL of fresh LB broth in a 1 L Erlenmeyer flask, which was incubated at the conditions described above. Next, the cellular suspension was centrifuged at 7871× *g* for 10 min, washed twice with sterile (121 °C and 15 psi for 15 min) saline solution (0.85 NaCl *w/v*), and the supernatant was discarded. Finally, the bacterial pellet was re-suspended in 200 mL sterile saline solution and adjusted to a concentration of ~10^9^ colony-forming units (CFU)·mL^−1^. Bacterial suspensions obtained through this method were used throughout the study.

### 4.2. Viability of PGPR Strains Exposed to Sodium Alginate

Four mL of each bacterial strain were re-suspended in sterile saline water to adjust the concentration to 1 × 10^4^ CFU·mL^−1^ and were individually poured into triplicate 125 mL Erlenmeyer flasks containing 21 mL of 2 % sodium alginate (MP Biomedicals, Cat. 154723) as the encapsulation material (or LB broth as control). The sodium alginate culture medium was prepared according to Rodrigues et al. [37]: 0.42 g of sodium alginate was dissolved in 21 mL of deionized water, homogenized at 40 °C, in a rotary shaker at 3× *g*, left to stabilize and hydrate overnight at room temperature, and autoclaved. Cultures were grown at 32 °C with shaking at 5× *g*, and aliquots were collected upon inoculation (0 min), and every 30 min until 180 min. CFU were determined by serial diluting and plate-count method on LB agar plates, incubated for 48 h under culture conditions described above. The doubling time (*t_d_*) was calculated by the following Equation (1):

(1) $$t=\frac{Ln\;\left(2\right)}{µ}$$

where *t* was the doubling time and *µ* was a specific growth rate.

### 4.3. Encapsulation of PGPR Strains

Bacterial strains were encapsulated in sodium alginate. For this, 200 mL of bacterial suspensions (10^9^ CFU·mL^−1^) of each strain were separately mixed with 800 mL of sterile high-viscosity sodium alginate (2%, 280 mPa) (MP Biomedicals, Cat. 154723), by stirring for 1 h at room temperature in an Erlenmeyer flask. Once each bacterial suspension was properly homogenized, the Erlenmeyer flask was connected to a microbeads-producing device [55], under aseptic conditions. The microbeads were created when the bacterial suspension was forced to pass through a 222 µm diameter nozzle with a pressure of 20 psi and sprayed into a stainless tray containing 1 L of sterile 2% CaCl_2_ solution, under agitation at 40 rpm. After 30 min of stirring in this solution [28], the microbeads were separated by filtration through Whatman no. 4 filter paper and washed three times with sterile saline solution. Next, the microbeads were placed in a stainless-steel tray covered with brown paper and dried in a convection oven at 34 °C for 24 h. Subsequently, the dried microbeads were transferred to a sterile hand mortar to pulverize the clumps and pass through a 200 µm sieve. Finally, the obtained microbeads were collected and maintained in sterile and hermetically sealed containers with silica gel [55]. The encapsulation yield (EY) was calculated by the following Equation (2) [61]:

(2) $$EY\;\left(\%\right)=\left(\frac{N}{N_{0}}\right)\times 100$$

where *N* was the total CFU encapsulated in microbeads and *N*_0_ was the CFU before encapsulation.

### 4.4. Viability of Encapsulated Strains

A modified method described by Galaviz et al. [62] was used to check the viability of encapsulated strains. For this, 50 mg of dry microbeads were dissolved in 15 mL of sterile citrate buffer (55 mM sodium citrate, 30 mM EDTA anhydride, and 150 mM NaCl_2_, adjusted to pH 8). The solution was shaken in a Vortex for ∼30 min until the microbeads were completely dissolved; then, the bacterial suspension was centrifuged at 7871× *g* for 10 min. Then, the supernatant was decanted, and the pellet was re-suspended in 1 mL 0.85% sterile saline solution, CFU were assessed by serial dilution and plating in LB agar (US Biological Cat. L1515). Triplicate samples of each strain were obtained every 3 months for 12 months (during this period the encapsulated strains were stored at room temperature).

### 4.5. Scanning Electron Microscopy

In addition, the morphological properties of microbeads were observed; for this, five microbeads were processed for observation by scanning electron microscopy (SEM) [63]. The microbeads were mounted on a stub and coated with gold film for 35 min at 40 mA in a sputter coater (Vacuum Desk II, Denton, Scotia, NY, USA). Visualization was done in an S-3000N microscope (Hitachi High-Technologies, Tokyo, Japan) at 15 kV, using a 45° angle of the slide to the electron beams. The photomicrographs were analyzed with the software Quartz PCI v5.5 (Quartz Imaging, Vancouver, BC, Canada).

### 4.6. Microbead Swelling, Biodegradation, and Bacterial Release-Establishment in Soil

Sterile soil (autoclaved five times at 121 °C and 15 psi pressure, for 1 h) previously collected in the Yaqui Valley [clay loam texture, organic matter content of 0.8%, pH of 7.9, major elements such as phosphorus (P), and nitrogen (N) was in a proportion of 50, and 139 Kg ha^−1^, respectively] [11] was used for these experiments. The swelling ratio (SR) of the microbeads in the soil was determined by placing one gram of microbeads into a nylon bag (*W_d_* = weight of nylon bags + dry microbeads) (described below); nylon bags were buried in 100 g sterile soil in sterilized hermetic containers (washed with 70% *vol/vol* ethanol, and UV light treatment for 1 h, 3 consecutive days). The containers were placed inside a growth chamber (BJPX-A450, BIOBASE) to simulate environmental conditions observed in the Yaqui Valley [13 h of darkness at 14 °C, 2 h of light at 18 °C, 7 h of light at 25 °C, and 2 h of light at 18 °C, based on records of REMAS weather station (27°22′12.28″ N and 109°55′51.71″ W) located at the Yaqui Valley], and the soil was water-saturated by adding sterile distilled water. After three days until one week, bags were carefully removed from the soil, the excess soil was removed, and bags were dried and weighed (*W_t_* = nylon bags + dry microbeads). The SR (%) of microbeads was determined by the following equation [51]. This experiment was carried out in triplicate.

(3) $$SR=\frac{W_{t}-W_{d}}{W_{d}}\;\times \;100\%$$

For the determination of biodegradation, 250 g of sterile soil was placed into sterilized hermetic containers sterilized as described above. Then, one gram of microbeads was placed into a nylon bag (2.5 × 1 cm) of fine mesh (~50 µm) and buried 5 cm below the soil surface. These hermetic containers were placed inside a growth chamber (BJPX-A450, BIOBASE) to simulate environmental conditions observed in the Yaqui Valley (described above) and were kept slightly below water saturation (~35%) by adding distilled water, as necessary. The initial weight of dried microbeads was recorded at the beginning of the experiment, and then the bags were removed from the soil and weighed every week for the following 30 days. Finally, microbeads in bags were examined under a stereoscopic microscope at the end of the experiment. All the experiments were carried out in triplicate [51,55].

The release-establishment of bacterial strains in soil was determined by the conventional plate count method on agar LB (US Biological Cat. L1515) complemented with the macro and microscopic growth characteristics related to the strains under study, previously reported [34]. For this, 1 g of soil was serially diluted up to 10^12^ and the CFU g dry soil^−1^ was determined in triplicate, every three days for 30 days [55].

### 4.7. Plant Growth Promotion by Encapsulated Strains under Growth Chamber Conditions

Wheat (*Triticum turgidum L.* subsp. *durum*) seeds var. CIRNO C2008 was used as a model plant to quantify the ability of encapsulated bacterial strains to promote the plant growth of wheat. Seeds (N = 250) were disinfected with 1.5% sodium hypochlorite (*v/v*) for 10 min, followed by three washes with sterile distilled water [34]. Disinfected seeds were germinated in rolled wet paper towels (50 × 25 cm) [64], and one seedling per pot was aseptically transferred to forest trays (diameter 4.7 cm), which contained 90 g of a mixture of sterile (five consecutive days at 121 °C, 15 psi for 1 h) soil and perlite (70:30 ratio). Then, two days after the microbeads were prepared, 0.001 mg (TRQ65), 0.02 mg (TRQ8), and 0.45 mg (TE3^T^) were taken (~10^7^ CFU g^−1^) and were applied to each planting hole in the potting soil, ensuring proximity between microbeads and roots. The soil was saturated with sterile water before sowing. Six treatments were evaluated, consisting of wheat seedlings with microbeads containing strain (1) TE3^T^, (2) TRQ8, (3) TRQ65, (4) TRQ8 + TRQ65, (5) TE3^T^ + TRQ8, and (6) a control treatment (empty microbeads). Pots were placed in a growth chamber (Conviron TC 16, Winnipeg, MB, Canada) under climatic conditions registered in the Yaqui Valley (described above), under a randomized experimental design. The treatments corresponding to the co-inoculation of strains TE3^T^ + TRQ65 and TE3^T^ + TRQ65 + TRQ8 were not carried out since these formulations have not shown positive effects on wheat biometric traits (stem and root length, biovolume index, stem, and root dry weight), under similar growth conditions, as previously reported by Rojas-Padilla et al. [34]. Two biological replicates per treatment, containing fifteen seedlings (*n* = 30 plants per treatment) were performed. The evaluated biometric parameters in wheat seedlings were stem length (SL), root length (RL), stem dry weight (SDW), and root dry weight (RDW) [56].

### 4.8. Plant Growth Promotion by Encapsulated Strains under Greenhouse Conditions

For greenhouse experiments, wheat seeds were germinated, disinfected, and transplanted as described above; then each was placed into pots containing 1.5 kg of solarized soil (15 days at ~30 °C). Then, ~10^7^ CFU g^−1^ of each *Bacillus* microbead was applied to seedlings (treatments evaluated mentioned above). This assay was carried out in duplicate (*n* = 30 plants per treatment), in two different agricultural seasons (2017–2018 and 2018–2019), under climatic conditions observed in the Yaqui Valley from December (sowing) to February (end of the experiment) (average temperature 17.2 °C, and humidity 68%). Wheat biometrics parameters measured at 65 days post-inoculation were stem diameter, biovolume index (circumference × stem length), stem and root length, dry weight of stem and root, and chlorophyll (SPAD Units) [11,56]. 

### 4.9. Statistical Analyses

Experimental data were analyzed separately. Plant growth data per experiment were analyzed using ANOVA and Tukey’s post hoc analysis at *p* ≤ 0.05 using Statistica v.10 (Tibco Software, Palo Alto, CA, USA).

## 5. Conclusions

The present study demonstrated that co-inoculation of alginate microbeads containing *Bacillus* strains showed a positive effect on wheat plants, and the encapsulation did not affect the plant growth-promoting activity of the studied bacteria. Encapsulation technology applied with calcium alginate protects bacterial cells from adverse conditions during storage and application in soil. In addition, the swelling allows bacteria to create release channels from the microbeads, and the biodegradation of microbeads enables the bacteria to establish themselves in the soil for a long time. Additionally, the microbeads inoculated into the soil could perform as a small bioreactor increasing the initial cell concentration.

## Figures and Tables

**Table 1:** Survival of *Bacillus* strains encapsulated in dry microbeads (*n* = 3). Data represent the mean.

Samples	Total Viable Cells in Suspension (CFU L^−1^)	Total Viable Cells after Encapsulation (CFU·g^−1^)	Total Grams of Obtained Microbeads	Total Viable Cells Encapsulated	Encapsulation Yield (Equation (1))
***B. cabrialesii* TE3^T^**	2.6 × 10^11^	2.4 × 10^10^	18.2 g	4.4 × 10^11^	162.4%
***B. megaterium* TRQ8**	4.4 × 10^12^	3.4 × 10^11^	17.5 g	6.0 × 10^12^	133.9%
***B. paralicheniformis* TRQ65**	5.7 × 10^12^	1.0 × 10^12^	18.3 g	1.8 × 10^13^	338.0%

**Table 2:** Wheat growth promotion by *Bacillus* strains in microbeads (individual or consortium) and control (empty microbeads) on biometric plant parameters, in two cycles of greenhouse assay.

Greenhouse Experiment	Bacterial Strain	Stem Length (cm)	Root Length (cm)	Stem Dry Weight (g)	Root Dry Weight (g)	Biovolume Index	Chlorophyll (SPAD Unit)
**Cycle 1 (2017–2018)**	TE3^T^	35.5 ± 0.9 b	32.3 ± 1.5 bc	0.72 ± 0.07 b	0.47 ± 0.05 ab	28.1 ± 1.1 c	46.1 ± 1.1 a
	TRQ65	41.4 ± 0.6 a	35.1 ± 0.9 ab	0.89 ± 0.04 a	0.33 ± 0.01 cd	35.2 ± 0.9 a	46.8 ± 1.1 a
	TRQ8	35.2 ± 0.8 b	36.1 ± 0.9 ab	0.63 ± 0.06 b	0.30 ± 0.02 d	30.4 ± 1.2 bc	43.9 ± 0.5 a
	TE3^T^ + TRQ8	37.3 ± 0.8 b	35.4 ± 0.9 ab	0.78 ± 0.06 ab	0.48 ± 0.02 a	31.7 ± 1.4 b	45.3 ± 0.9 a
	TRQ8 + TRQ65	39.4 ± 0.9 a	36.9 ± 1.0 a	0.79 ± 0.07 ab	0.36 ± 0.03 c	34.0 ± 1.6 ab	45.1 ± 1.6 a
	CONTROL	36.1 ± 1.9 b	29.9 ± 0.9 c	0.69 ± 0.07 b	0.42 ± 0.04 b	32.8 ± 2.1 b	46.6 ± 0.6 a
**Cycle 2 (2018–2019)**	TE3^T^	44.7 ± 1.5 c	35.9 ± 1.5 c	1.0 ± 0.10 c	0.42 ± 0.03 d	40.2 ± 2.3 a	49.3 ± 0.5 ab
	TRQ65	47.9 ± 1.0 bc	44.4 ± 0.8 a	1.8 ± 0.15 a	0.55 ± 0.05 b	45.4 ± 2.1 a	48.0 ± 0.5 bc
	TRQ8	51.4 ± 0.9 ab	38.2 ± 2.5 b	1.5 ± 0.07 b	0.43 ± 0.02 d	43.0 ± 1.3 a	50.4 ± 0.4 a
	TE3^T^ + TRQ8	51.1 ± 0.9 ab	37.1 ± 2.5 bc	1.1 ± 0.05 c	0.49 ± 0.03 c	42.5 ± 1.2 a	50.5 ± 0.5 a
	TRQ8 + TRQ65	53.7 ± 0.9 a	44.2 ± 1.2 a	1.5 ± 0.09 b	0.67 ± 0.06 a	46.7 ± 1.7 a	50.1 ± 0.4 a
	CONTROL	49.1 ± 1.0 bc	40.2 ± 1.5 b	1.4 ± 0.10 b	0.54 ± 0.05 b	46.2 ± 1.5 a	46.8 ± 0.6 c

Means (*n* = 15) with the same letter are not significantly different, according to the Tukey–Kramer test (*p* ≤ 0.05).

## Data Availability

Data is contained within the article.
